# Structural Characterization of Act c 10.0101 and Pun g 1.0101—Allergens from the Non-Specific Lipid Transfer Protein Family

**DOI:** 10.3390/molecules26020256

**Published:** 2021-01-06

**Authors:** Andrea O’Malley, Swanandi Pote, Ivana Giangrieco, Lisa Tuppo, Anna Gawlicka-Chruszcz, Krzysztof Kowal, Maria Antonietta Ciardiello, Maksymilian Chruszcz

**Affiliations:** 1 Department of Chemistry and Biochemistry, University of South Carolina, Columbia, SC 29208, USA; aomalley@email.sc.edu (A.O.); spote@email.sc.edu (S.P.); gawlicac@mailbox.sc.edu (A.G.-C.); 2 Division of Naples, Institute of Biosciences and BioResources, National Research Council, Via Pietro Castellino, 111, 80131 Napoli, Italy; ivana.giangrieco@ibbr.cnr.it (I.G.); lisa.tuppo@ibbr.cnr.it (L.T.); 3 Allergy Data Laboratories (ADL), Via Nino Bixio, 11, 04100 Latina, Italy; 4 Department of Allergology and Internal Medicine, Medical University of Bialystok, 15-276 Bialystok, Poland; kowalkmd@umb.edu.pl; 5 Department of Experimental Allergology and Immunology, Medical University of Bialystok, 15-276 Bialystok, Poland

**Keywords:** food allergy, nsLTP, cross-reactivity, structure, natural source

## Abstract

**(1) Background:** Non-specific lipid transfer proteins (nsLTPs), which belong to the prolamin superfamily, are potent allergens. While the biological role of LTPs is still not well understood, it is known that these proteins bind lipids. Allergen nsLTPs are characterized by significant stability and resistance to digestion. **(2) Methods:** nsLTPs from gold kiwifruit (Act c 10.0101) and pomegranate (Pun g 1.0101) were isolated from their natural sources and structurally characterized using X-ray crystallography **(3) Results:** Both proteins crystallized and their crystal structures were determined. The proteins have a very similar overall fold with characteristic compact, mainly α-helical structures. The C-terminal sequence of Act c 10.0101 was updated based on our structural and mass spectrometry analysis. Information on proteins’ sequences and structures was used to estimate the risk of cross-reactive reactions between Act c 10.0101 or Pun g 1.0101 and other allergens from this family of proteins. **(4) Conclusions:** Structural studies indicate a conformational flexibility of allergens from the nsLTP family and suggest that immunoglobulin E binding to some surface regions of these allergens may depend on ligand binding. Both Act c 10.0101 and Pun g 1.0101 are likely to be involved in cross-reactive reactions involving other proteins from the nsLTP family.

## 1. Introduction

Lipid transfer proteins (LTPs), also called non-specific lipid transfer proteins (nsLTPs), are known for their ability to bind and transport hydrophobic ligands [1,2,3,4]. The biological role of nsLTPs is still not well understood. They transport these lipids between biological membranes primarily for defense, including structural adaptation, antimicrobial activity, and pathogenic resistance [5,6]. They are ubiquitous proteins found in plants, microorganisms, and animals, and they are included in the broader panallergen classification [7,8,9]. Plant nsLTPs are divided into two main subfamilies according to their molecular masses: 9-kDa and 7-kDa LTPs [10]. Members of both the subfamilies are highly resistant to heat and denaturation [11,12]. This high stability is associated with a mainly helical compact structure stabilized by four disulfide bonds [13,14]. Despite similar overall structures, 9-kDa and 7-kDa LTPs share relatively low sequence identity and the pattern of cysteine pairing in the formation of disulfide connectivity is different [15]. Furthermore, 7-kDa LTPs lack some regions found in 9-kDa LTPs, including large fragments in the central and C-terminal parts of the molecule [10].

nsLTPs comprise an important group of panallergens that originate from plants [16,17]. Pru p 3, the 9-kDa LTP from peach, is considered to be one of the most important allergens from this family, and was shown to be of special interest in Mediterranean countries [3,18]. In Italy, it was reported that nsLTPs are the most frequent sensitizer in individuals affected by food-dependent exercise-induced anaphylaxis [19]. Sensitization to Pru p 3 may cause allergic reactions to other fruits and seeds such as apple (Mal d 3), cherry (Pru av 3), gold kiwi (Act c 10), green kiwi (Act d 10), orange (Cit s 3), and pomegranate (Pun g 1) because of cross-reactivity events. Cross-reactions between allergens represent a significant risk for allergic people, and they are caused by the immunoglobulin E (IgE) epitopes shared at least partially between homologous proteins which are co-recognized by specific antibodies. The competition for IgE binding displayed by allergens with shared epitopes has been widely exploited to investigate cross-reactions between homologous allergens, including nsLTPs.

The IgE-binding epitopes found in Pru p 3 are similar to those found in other nsLTP allergens, giving an explanation for reported cross-reactivity [20]. Since the major IgE-binding epitopes in such allergens are close to the lipid-binding region, it is likely that lipid binding affects allergenicity [14]. Because of the inherent heat- and protease-resistance of nsLTPs, ingestion may cause systemic class I allergic reactions, encompassing symptoms such as urticaria, angioedema, gastrointestinal pain, and anaphylaxis [14,21]. Clinically, allergy to nsLTPs is highly diverse, ranging from mild, local symptoms such as oral allergy syndrome (OAS) to very severe, life-threatening systemic anaphylaxis [22]. In patients allergic to nsLTPs from some plants, tolerance to nsLTPs from other plants is seen [18]. Sensitization to nsLTPs from many plants is usually associated with more severe clinical response [23]. Moreover, the type of clinical response may depend on the localization of an individual protein within a given fruit or a vegetable or on the physicochemical properties of those proteins [22].

Thirteen kiwifruit allergens originating from *Actinidia deliciosa* (green kiwifruit) and four from *Actinidia chinensis* (gold kiwifruit) have been registered by the World Health Organization and International Union of Immunological Societies (WHO/IUIS) Allergen Nomenclature Sub-committee [24]. Kiwifruit allergens are relatively well characterized [25,26,27,28,29,30]; however, only a small fraction of these have their structures determined [31,32,33,34]. In contrast, there are only three allergens [21,35,36] originating from pomegranate that are registered by WHO/IUIS, only one of which, chitinase III (Pun g 14), has its structure determined [37]. However, Pun g 14 seems to be a minor allergen in pomegranate, whereas Pun g 1 is reported as a major allergen with a sensitization prevalence of almost 60%, as reported for a population of 357 patients with IgE positive to this fruit [36]. Similarly, while kiwifruit allergy has been recognized for a relatively long time [38], allergy to pomegranate has only recently been recognized as important, which is due to increased consumption of this fruit [39].

We elucidated the crystal structures of Act c 10.0101 and Pun g 1.0101, nsLTP allergens from gold kiwifruit and pomegranate, respectively. These allergens both belong to the subfamily of 9-kDa LTPs. Structural studies highlight the conformational flexibility of both proteins and provide hints on their interactions with lipids. Moreover, we have analyzed Act c 10.0101 and Pun g 1.0101 structures in connection to their potential to participate in cross-reactive allergic reactions.

## 2. Results

### 2.1. Overall Structures of Act c 10.0101

**Figure 1:**
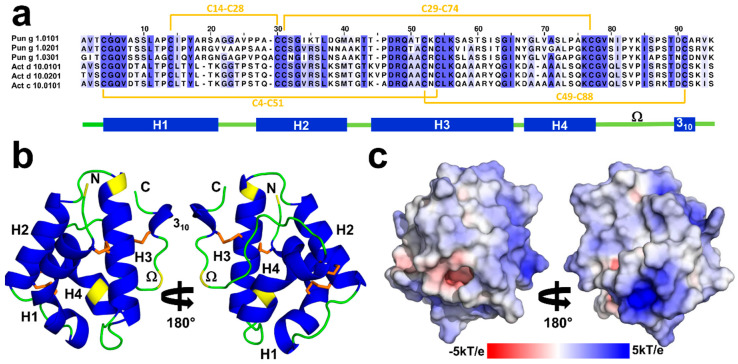
(**a**) Sequence alignment of non-specific lipid transfer proteins (nsLTPs) originating from pomegranate and kiwifruits. Disulfide bridges observed in Act c 10.0101 are marked in orange. Secondary structure elements are indicated below the sequence alignment. Helices are shown in blue and loops in green. Yellow color indicates residues that are different between Act c 10.0101 and Act d 10.0101 or Act d 10.0201. (**b**) Cartoon representation of Act c 10.0101 shown in two different orientations. Disulfide bridges are marked in orange. (**c**) Surface representation of Act c 10.0101 showing distribution of charges.

Act c 10.0101 crystallized in the P2_1_ space group with two protein molecules in the asymmetric units. The model was refined to 1.95Å resolution. The crystal structure corresponds to a closed apo form of the protein. Both protein chains include residues 1–92 (mature form of the protein) that are folded into a compact, mainly α-helical structure that is characteristic for nsLTPs (Figure 1). The protein chains adopt very similar conformations and they superposed with Root Mean Square Deviation (RMSD) value of 0.3Å over 92 Cα. The Act c 10.0101 structure is stabilized by four disulfide bridges that are formed by the following pairs of cysteine residues: Cys4–Cys51, Cys14–Cys28, Cys29–Cys74, and Cys49–Cys88 (Figure 1).

According to PDBePISA [40], the protein chains present in the asymmetric unit may form a stable dimer. However, the buried surface area between molecules forming the crystals is approximately 580 Å^2^, which is significantly below the cutoff value of 856 Å^2^ that was proposed for discrimination between homodimeric and monomeric proteins by Ponstingl et al. [41]. The gel filtration results indicate that both Act c 10.0101 and Pun g 1.0101 are monomeric in solution (Supplementary Figure S1). Moreover, these results show that the proteins are monomeric in solutions with pH = 8, as well as pH = 4.5, which corresponds to crystallization conditions and pH characteristic to kiwifruit and pomegranate.

**Figure 2:**
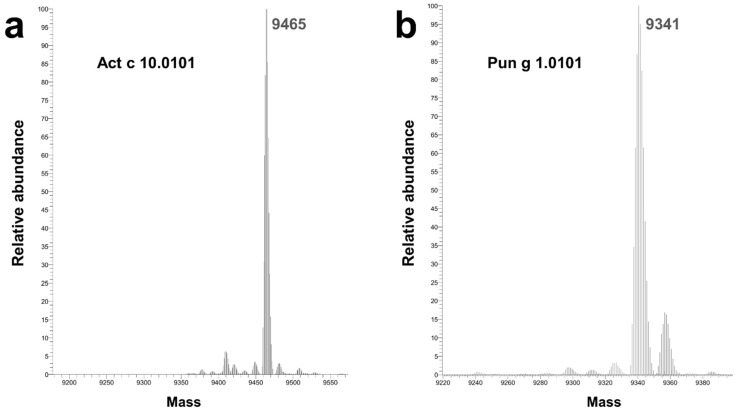
Results of mass spectrometric analysis of (**a**) Act c 10.0101 and (**b**) Pun g 1.0101 samples used for crystallization experiments. Calculated molecular weights for Act c 10.0101 and Pun g 1.0101 that have intact disulfide bridges are 9453 and 9342 Da, respectively. When there is no formation of disulfide bridges, the calculated molecular weights are 9461 and 9350 Da for Act c 10.1010 and Pun g 1.0101, respectively.

The sequence of the Act c 10.0101 that we have crystallized differs slightly from one that was previously reported (UniProt entry P85204). We have identified three additional amino acids at the C-terminal end of the protein and modeled these amino acids as Lys-Ile-Ser, which is consistent with the sequence that is observed in homologous Act d 10.0101 and Act d 10.0201 (Figure 1), as well as in agreement with the results of mass spectrometric analysis of the sample used for crystallization (Figure 2). The crystal structure revealed that Lys90 is solvent exposed, the side chain of Ile91 points toward the hydrophobic core of the protein, and the C-terminal carboxyl group of Ser92 forms a salt bridge with Arg45.

The comparison of the primary structure of Act c 10.0101 with the homologous allergens from green kiwifruit shows the substitution of three internal residues. In fact, only Ser10, Ala43, A66, and Lys85 of Act c 10.0101 are not conserved in Act d 10.0101 and Act d 10.0201 (Figure 1). In addition, Act c 10.0101 shares the N-terminal amino acid (Ala1) with Act d 10.0101, whereas in Act d 10.0201, this residue is substituted with Thr1. Therefore, the sequence identities between the gold kiwifruit nsLTP and Act d 10.0101 and Act d 10.0201 are 97% and 96%, respectively.

### 2.2. Overall Structures of Pun g 1.0101

**Figure 3:**
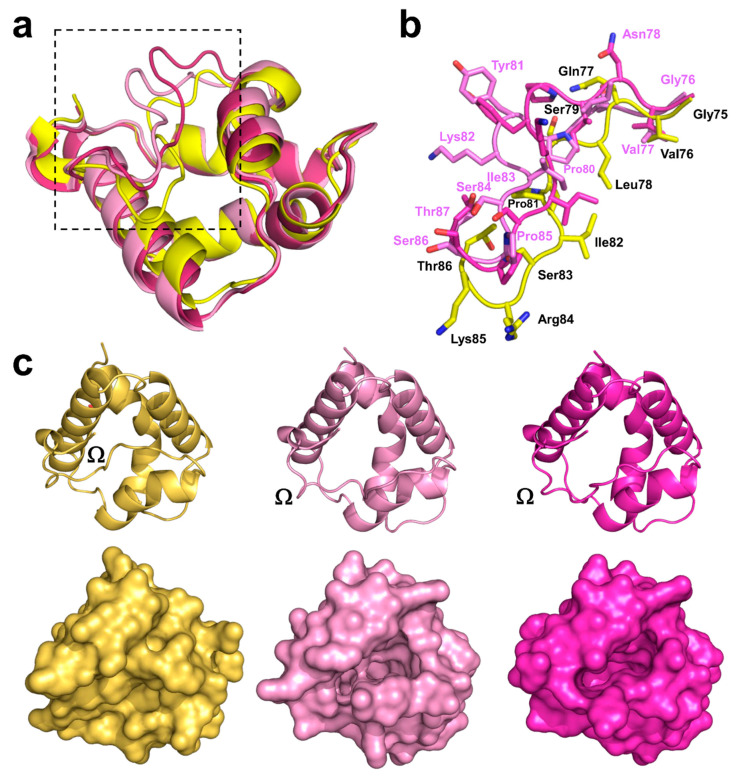
(**a**) Superposition of Act c 10.0101 and Pun g 1.0101 models shown in cartoon representation. Act c 10.0101 is shown in yellow, while Pun g 1.0101 models corresponding to two different conformations of the molecules are presented in different shades of pink. Regions of the proteins showing the biggest changes in conformation correspond to the Ω loops and were marked with the black square. (**b**) Close-up view of the Ω loops. Residues from Act c 10.0101 are marked using black labels, while Pun g 1.0101 residues are labeled in pink. (**c**) Models of Act c 10.0101 and Pun g 1.0101 in cartoon (top) and surface (bottom) representations. The models are shown in an orientation that shows the role of the Ω loops in closing and opening access to the ligand binding cavity.

Pun g 1.0101 also crystallized in the P2_1_ space group; however, the asymmetric unit contains four protein molecules. The model was refined to 2.4Å resolution. The protein molecules forming the crystal have the sequence that corresponds to Pun g 1.0101, and all four chains include all 93 residues. Similar to Act c 10.0101, the structure of Pun g 1.0101 is stabilized by four disulfide bridges formed by residues Cys4–Cys52, Cys14–Cys29, Cys30–Cys75, and Cys50–Cys89. However, the conformations of the protein molecules present in the asymmetric unit differ, and half of the molecules have a distinct conformation corresponding to residues 77–85. This stretch of the polypeptide chain controls the opening of the ligand binding cavity [42] and corresponds to the Ω loop (Figure 1). The conserved Cys75 and Cys89 can be treated as “anchor points” for the loop. Comparison of Act c 10.0101 and Pun g 1.0101 structures suggests that conserved Asn78 and Thr87 may play the role of “hinges” that allow the Ω loops to change the conformation (Figure 3b). Chains A and B adopt one conformation, and chains C and D adopt the second observed conformation. Despite the different conformations, none of the protein chains have a ligand bound; therefore, the crystal structure provides insight into the structure of two Pun g 1.0101 apo forms. The protein chains that have different conformation of the 77–85 region superpose with RMSDs of 0.9 Å; at the same time, chains with the same conformations overlap with RMSDs of approximately 0.3 Å. While our structure of Act c 10.0101 corresponds to a closed conformation of the protein, both conformations of Pun g 1.0101 correspond to at least partially open structures (Figure 3). In the case of chains A and B, cavities with volumes of approximately 370 and 340 Å^3^ are observed, and in the case of chains C and D, the cavity volume is ~414 Å^3^. This is significantly less in comparison with lentil nsLTP (Len c 3.0101) for which it was shown that the cavity expanded from ∼600 to ∼1000 Å^3^ after ligand binding [43,44].

Similar to Act c 10.0101, PDBePISa suggests the possibility for potentially stable dimeric assemblies in the chains of the crystal structure for Pun g 1.0101. It is suggested that chains A and C, as well as chains B and D, form dimers with interface areas of 615 and 618 Å^2^, respectively. However, these values are again significantly below the cut-off previously proposed for homodimeric proteins, and the gel filtration results confirmed that Pun g 1.0101 is monomeric in solution (Figure S1).

### 2.3. Structural Comparison with Other nsLTPs

#### 2.3.1. Overall Protein Structure

Act c 10.0101 and Pun g 1.0101 share the same overall structure and have 45% sequence identity. Sequence- and structure-based searches against the PDB revealed several plant nsLTPs that are similar to Act c 10.0101 (sequence identity lower than 53%) and Pun g 1.0101 (sequence identity lower than 67%). The most similar to Act c 10.0101 (RMSD values ~1.2–1.6Å) are structures of nsLTPs from eggplant (PDB code: 5TVI), lentil (PDB code 2MAL), rice (PDB code: 1BV2), maize (PDB code: 1AFH), peach (PDB code: 2ALG), wheat (PDB code: 1BWO), dill (PDB code: 2N2Z), barley (PDB code: 1BE2), and mugwort (PDB code: 6FRR). The same proteins also appear in comparison to Pun g 1.0101, as well as nsLTPs from tobacco (PDB code: 1T12), hazelnut (PDB code: 4XUW), pea (PDB code 2N81), and mung bean (PDB code 1SIY). All mentioned nsLTPs have the conserved pattern of disulfide bridges and the same arrangement of secondary structure elements. Moreover, these proteins are reported as thermostable and resistant to digestion [45,46,47]. They are all monomeric with high pI values and significant content of Lys and Arg residues.

**Figure 4:**
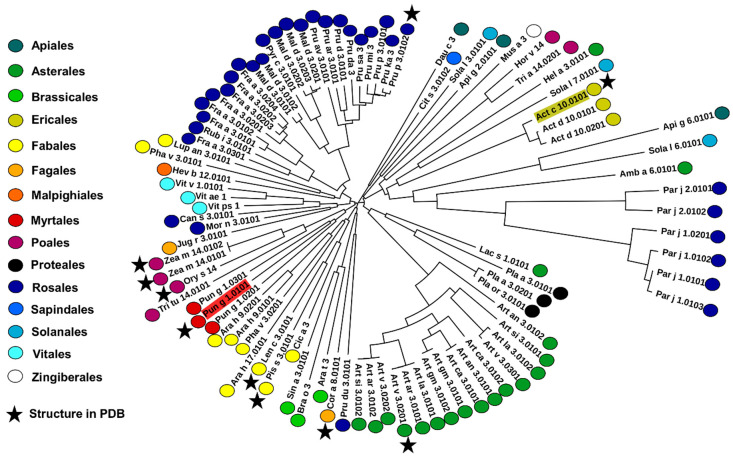
Phylogenetic tree constructed with sequences of nsLTPs that are reported as allergens. Dots next to proteins’ names indicate orders from which particular allergens originate. Stars mark proteins that have their structures determined and reported to the Protein Data Bank [51].

A significant fraction of these proteins is reported as allergens, with Pru p 3 (9-kDa LTP from peach) being a representative and important allergenic molecule [20,48,49,50]. However, among over 90 allergens and isoallergens, very few have their structures determined (Figure 4), and Act c 10.0101 and Pun g 1.0101 represent proteins that are not yet well experimentally characterized. Several plant orders from which allergenic nsLTPs originate currently do not have any representative structures. Our structures of Act c 10.0101 and Pun g 1.0101 are the first representative structures of allergenic nsLTPs from Ericales and Myrtales, respectively (Figure 4).

#### 2.3.2. Ligand Binding

**Figure 5:**
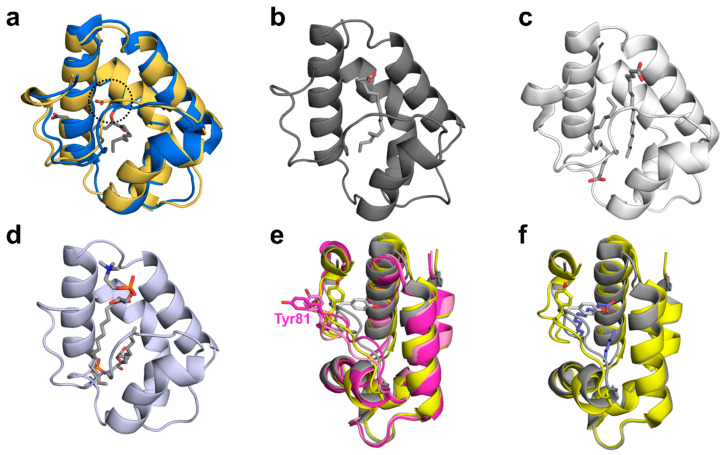
Lipid binding modes observed in nsLTPs. (**a**) Covalent binding of oxylipin by barley nsLTP (PDB code: 3GSH). Model of barley nsLTP is shown in blue. Structure of Act c 10.0101 is shown in gold. Asp7 (barley), Asp8 (Act c 10.0101), and oxylipin are shown in stick representation. Position of the conserved Asp is marked with a circle. (**b**) Complex of maize nsLTP with α-linolenic acid (PDB code: 1FK6). (**c**) Complex of rice nsLTP with two molecules of palmitoleic acid (PDB code: 1UVB). (**d**) Complex of wheat nsLTP with two molecules of lyso-myristoyl-phosphatidylcholine (PDB code: 1BWO). (**e**) Superposition of Cor a 8 (PDB code: 4XUW; grey), Pru p 3 (PDB code: 2ALG; yellow), and models corresponding to two different conformations of Pun g 1.0101 (pink). Conserved Tyr81 is shown in stick representation. (**f**) Superposition of Cor a 8 and Pru p 3. Lipids bound by Pru p 3 are shown in purple. The figure shows that the conformation of the conserved residues that is present in the Cor a 8 structure is not compatible with ligand binding.

Among reported plant nsLTPs structures, there are many that illustrate interactions of this group of proteins with hydrophobic molecules. These structures also provide insight into the flexibility of nsLTPs and their ability to change conformation in order to bind ligands (Figure 3 and Figure 5). The structures shown in Figure 5 illustrate 1:1 or 1:2 stoichiometry for nsLTP binding of lipids. Moreover, they show that, while in most cases, the binding involves only weak interactions, in some cases, formation of a covalent bond between the protein and lipid is observed. The covalent ligand binding was shown in the case of oxylipin-conjugated barley 9-kDa LTP (Figure 5a), where Asp7 of this nsLTP is responsible for the formation of the bond between the protein and lipid. This aspartic acid residue is conserved in Act c 10.0101 (Asp8), Act d 10.0101, Act d 10.0201, chickpea Cic a 3, tomato Sola l 7.0101, and wheat Tri a 14.0201, which suggests that these nsLTPs may have a similar mode of ligand binding and may also bind oxylipins. While the aspartic acid forming the covalent bond is not conserved in Pun g 1.0101, it is likely that this protein may bind oxidized lipids with a different mode, such as that observed in the case of maize nsLTP (Zea m 14) complexed with ricinoleic acid (PDB code: 1FK7) [52]. The nsLTPs that have the conserved aspartic acid residue also may form noncovalent complexes with lipids, which is illustrated by the structure of Tri a 14.0201 in complex with a phospholipid (Figure 5d; PDB code: 1BWO) [53]. This structure and the structure of rice nsLTP (Ory s 14; PDB code: 1UVB) provide examples of complexes between nsLTPs with two lipid molecules (Figure 5c,d).

The difference in likely modes of ligand binding for Act c 10.0101 is not only related to the presence of the conserved aspartic acid residue in kiwifruit protein, but also the absence of a tyrosine residue in the Ω loop (Figure 1, Figure 3 and Figure 5). This tyrosine (residue 81) is present in Pun g 1.0101 isoallergens, and it was shown to significantly change conformation after ligand binding. For example, in the structure of *apo* Cor a 8 (nsLTP from hazelnut; PDB code: 4XUW), an equivalent tyrosine is facing toward the interior of the ligand biding cavity; in the structure of a complex between Pru p 3 and lipids (PDB code: 2B5S), this residue is facing toward the exterior of the protein (Figure 5e) [3,46]. On the other hand, the interiors of Act c 10.0101 and Pun g 1.0101 are very similar and are composed mainly of aliphatic amino acids that are most likely responsible for interactions with aliphatic chains of lipids.

### 2.4. Analysis of Potential Cross-Reactivity between Act c 10.0101, Pun g 1.0101, and Other nsLTPs

**Figure 6:**
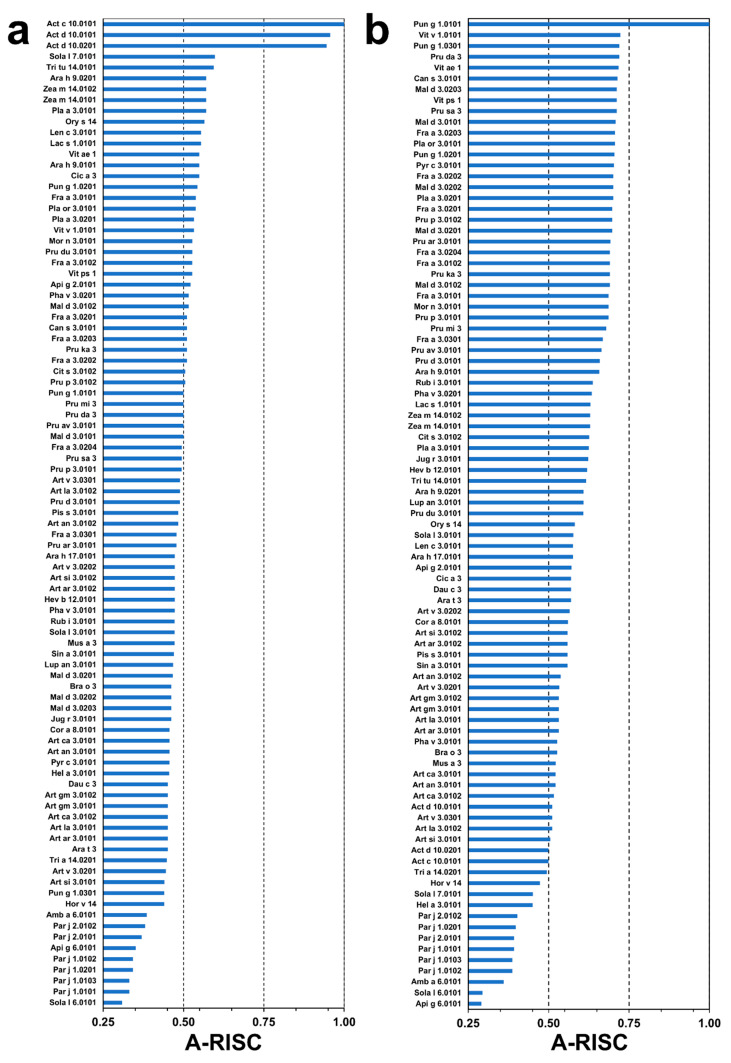
Plots showing Allergens’-Relative Identity, Similarity and Cross-reactivity (A-RISC) indexes of allergens from the nsLTP family. (**a**) Comparison of the family members to Act c 10.0101 and (**b**) Pun g 1.0101.

Act c 10.0101 is very similar in terms of sequence to Act d 10.0101 and Act d 10.0201, as these proteins share 95% of identical residues (Figure 1). To estimate the likelihood of cross-reactive reaction between Act c 10.0101 and other nsLTPs, we have used the following categories that were previously proposed: high (Allergens’-Relative Identity, Similarity and Cross-reactivity index (A-RISC) values ≥ 0.75), medium-high (0.75 > A-RISC ≥ 0.50), medium-low (0.50 > A-RISC ≥ 0.25), and low (A-RISC values < 0.25). A-RISC-based analysis of possible cross-reactivity between Act c 10.0101 and other allergens belonging to the nsLTP family indicates tomato Sola l 7.0101, durum wheat Tri tu 14.0101, peanut Ara h 9.0201, Zea m 14.0102, and Zea m 14.0101 as the most likely candidates for a cross-reactive reaction. A-RISC index values for these allergens are not higher than 0.6 and they fall into the medium-high risk cross-reactive reaction category (Figure 6a). Generally, a risk of cross-reactivity between kiwifruit nsLTPs and other allergenic proteins from this family is medium. The lowest likelihood of cross-reactive reactions with Act c 10.0101 is predicted for ragweed Amb a 6.0101, celery Api g 6.0101, Sola l 6.0101, and nsLTPs from *Parietaria judaica*. Api g 6.0101 and Sola l 6.0101 are classified as 7-kDa LTPs, and allergens from *P. judaica* not only have a relatively low sequence identity with Act c 10.0101, but they have an additional stretch of amino acids at their C-terminal ends.

Pun g 1.0101 shares 69% sequence identity with Pun g 1.0301 and 68% sequence identity with Pun g 1.0201. Therefore, it is not surprising that there is a relatively high likelihood of cross-reactive reactions caused by these allergens (Figure 6b). Estimation of likelihood of cross-reactive reactions between Pun g 1.0101 and other nsLTPs reveals that this allergen has a significantly higher potential to participate in such reactions when compared with Act c 10.0101. There are many allergens from nsLTP family for which the A-RISC value is close to 0.70 and therefore, approach the high-risk cross-reactivity likelihood category. Our estimation suggests that nsLTPs from grape (Vit v 1.0101), David’s peach (Pru da 3), American grape (Vit ae 1), Indian hemp (Can s 3.0101), apple (Mal d 3), strawberry (Fra a 3), pear (Pyr c 3.0101), peach (Pru p 3.0102), London plane tree (Pla a 3.0201), and Oriental plane tree (Pla or 3.0101) are most likely to be involved in cross-reactive reactions with Pun g 1.0101. Comparison of A-RISC profiles of Act c 10.0101 (Figure 6a) and Pun g 1.0101 (Figure 6b) clearly indicates that there is a significant difference in potential of both allergens for their cross-reactivity. However, in both cases, Amb a 6.0101, Api g 6.0101, Sola l 6.0101, and nsLTPs from *P. judaica* are the least likely candidates for cross-reactive reactions.

## 3. Discussion

Two allergens, Act c 10.0101 and Pun g 1.0101, were successfully purified from their natural sources and used for structural studies. Act c 10.0101 was purified from gold kiwifruit seeds, and it was never detected in the fruit pulp [25]. In contrast, Pun g 1.0101 was purified from pomegranate red pulp [21,36] and it has never been reported in the fruit seeds. The structural studies together with mass spectrometry confirmed the amino acid composition of Pun g 1.0101 and revealed the presence of three additional amino acids (Lys-Ile-Ser) at the C-terminal end of Act c 10.0101. The same C-terminal sequence is also observed in Act d 10.0101 and Act d 10.0201 (Figure 1).

Structural studies revealed that both Act c 10.0101 and Pun g 1.0101 share the same overall fold as other members of the nsLTP family, as well as the pattern of four disulfide bridges that stabilize three dimensional structures of these relatively small proteins. The structures we determined correspond to *apo* forms of the proteins. While the structure of Act c 10.0101 represents a closed conformation of the molecule, the crystal structure of Pun g 1.0101 reveals two different protein conformations with a partially open ligand binding cavity. Residues forming the nsLTP Ω loop display the most significant changes of conformations when comparing closed and open forms of the proteins. In Pun g 1.0101 but not in Act c 10.0101, this loop contains a conserved Tyr81 (Figure 1, Figure 3 and Figure 5) that was shown to change conformation depending on the presence or absence of ligands [3,46]. At the same time, this residue is not present in nsLTPs from kiwifruits, which suggests a difference in lipid binding modes between Act c 10.0101 and Pun g 1.0101.

Comparison of the determined structures with structures of nsLTPs in complexes with different lipids suggests that Act c 10.0101 may bind oxylipin similarly to barley nsLTP. The binding of oxylipin in the case of barley protein involved Asp7, which is also conserved in the kiwifruit protein (Asp8; Figure 5). In plants, an activation of oxylipin biosynthesis is initiated upon wounding or herbivory and pathogen attacks [54]. In humans, oxylipins are involved in inflammation, immunity, and vascular functions, and approximately one hundred of these compounds were identified [55]. Many oxylipins are mobilized during intensive and prolonged exercise. Therefore, we speculate that the ligand binding properties of allergens from the nsLTP family and the molecular cargo transported by these proteins may be associated with exercise-induced allergic reactions including anaphylaxis. This speculation is consistent with the report that nsLTPs are the most frequent sensitizer in individuals affected by food-dependent exercise-induced anaphylaxis [19]. Pun g 1 was also implicated as the cause of such reaction in a patient from Northern Italy [56].

Our analysis based on the A-RISC index shows that Act c 10.0101 and Pun g 1.0101 have different likelihoods for cross-reactive allergic reactions. A-RISC predicts a high level of cross-reactions between Act c 10.0101 and the homologous allergens from green kiwifruit, Act d 10.0101 and Act d 10.0201. This observation is confirmed by immunological and clinical studies showing a very similar behavior for all the kiwifruit nsLTPs [25]. In fact, a study focused on a population of patients sensitized to plant nsLTPs showed high IgE value correlations between gold and green kiwifruit nsLTPs (the latter tested as a mix of both the isoforms, Act d 10.0101 and Act d 10.0201). In addition, many subjects sensitized to Act c 10.0101 proved to be IgE positive to the green kiwifruit nsLTPs as well. Nevertheless, in vivo tests sometimes showed a non-completely overlapping behavior of green and gold kiwifruit nsLTPs. Similarly, it was observed that a few subjects (4.3% of 346 patients) were IgE positive to Act c 10.0101 and negative to Act d 10 isoforms [25], thus suggesting differences in the structure of one or more IgE binding epitopes of these molecules. Since the structural differences between green and gold kiwifruit nsLTPs could rely on four amino acid substitutions, namely Ser10, Ala43, Ala66, and Lys85 (Figure 1 and Supplementary Figure S2), it is conceivable that the different immunological behavior is correlated with these residues, as Ala43 and Lys85 are found on the molecule surface (Figure S2). In addition, it must be noted that there is also a deletion and insertion of an amino acid next to residues Ala43 and Ala66, which may significantly affect the structure of the protein in these areas. We suggest that one or more of these three residues belong to IgE epitopes and affect the interaction with antibodies. Actually, Ala43 falls in a region reported to be an important conformational and sequence epitope (epitope 2) in the peach nsLTP, Pru p 3 [10,57,58], the most powerful component of this allergen family. The residue Ala66 is found in a region that does not represent an IgE epitope in Pru p 3. Therefore, if the location of epitopes is conserved in allergenic nsLTPs, it is possible that the substitution of the residue in position 66 would not have any effect on the IgE binding of these allergens. Conversely, the residue in position 85 (Lys85 in Act c 10.0101) is included in a sequence epitope (epitope 3) in Pru p 3 [10,59]. Therefore, at least two regions corresponding to epitope 2 and epitope 3 in Pru p 3 could also be involved in IgE binding in Act c 10.0101. Each one of these regions in Act c 10.0101 shows a residue substitution that could be responsible for differences in IgE binding compared to Act d 10. It seems that especially the insertion of Ala66 may significantly change the distribution of charges on the protein surface, and the comparison of Act c 10.0101 and Act d 10.0101 models (Figure S2b).

**Figure 7:**
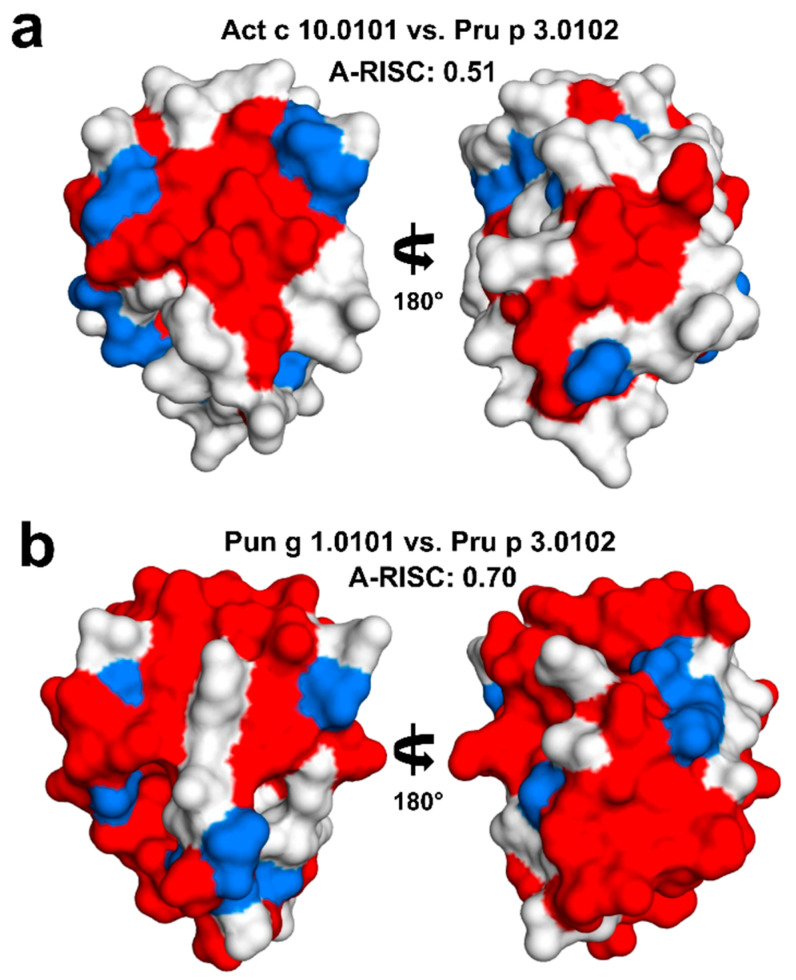
Surface residue conservation between Act c 10.0101 and Pru p 3.0102 (**a**), as well as between Pun g 1.0101 and Pru p 3.0102 (**b**). Identical residues are marked in red, while similar residues are marked in blue.

Data obtained by IgE inhibition experiments also confirmed the very high level of cross-reactions between nsLTPs from green and gold kiwifruit. In line with A-RISC predictions, kiwifruit nsLTPs are reported to show high levels (≥50%) of IgE-binding inhibition on the peanut Ara h 9, whereas lower values were observed on other allergenic nsLTPs, such as the peach Pru p 3, the hazelnut Cor a 8, and the pollen nsLTP Art v 3 [25]. The sequence similarity- and identity-based A-RISC index strongly suggests that Pun g 1.0101 is significantly more likely to participate in such reactions in contrast to Act c 10.0101 or Act d 10.0101 and Act d 10.0201 (Figure 6). The structures of Act c 10.0101 and Pun g 1.0101 allow for mapping of surface-exposed residues that are conserved between nsLTPs and form large patches on the surface that are identical or very similar and therefore, can be responsible for the binding of cross-reactive antibodies. The mapping of the conserved residues clearly indicates that one should expect a significantly higher likelihood of a cross-reactive reaction involving Pun g 1.0101 and Pru p 3.0102 in comparison with Act c 10.0101 and Pru p 3.0102 (Figure 7).

Our predictions based on structural similarities agree well with reported clinical cross-reactivity. It was shown that pomegranate nsLTPs are cross-reactive with Pru p 3, and the cross-reactivity may lead to anaphylactic shock [60]. Some patients with allergy to Pru p 3 also have IgE reactivity towards nsLTPs from kiwifruit [25]. However, the observation of at least partial IgE co-recognition between nsLTPs with low similarities, such as the 7-kDa tomato LTP (Sola l 6) and the 9-kDa LTPs including the kiwifruit homologs [10], suggests that additional factors could make the prediction of the IgE binding and IgE co-recognition and cross-reactions more complex than expected. For instance, the type of ligand binding of the allergenic protein can have some effects. nsLTPs bind lipids that, in addition to their role as direct immune modulators, could influence the allergenicity of a protein by modifying the allergen structure and biochemical properties [61]. In this context, a recent study demonstrated that the interaction of Pru p 3 with selected fatty acids increased its IgE binding capacity, whereas the binding of some other lipids did not have the same effect [62]. The ligand binding properties of nsLTPs cannot be investigated on the basis of the primary structure only. The availability of the three-dimensional structures of these allergens appears of utmost importance to investigate the features of the ligand binding cavity and to predict the possible ligands. This kind of information can contribute to understand the allergenicity, cross-reactions, and antigenic potency of the components of this protein family. The structures of Act c 10.0101 and Pun g 1.0101 reported here can provide a contribution in this direction. Although they were crystallized as *apo*-proteins, they show ligand binding cavities with different properties, thus suggesting they bind different ligands in vivo. Additional studies are needed to investigate a possible correlation between the structural, ligand binding, and IgE binding properties of these proteins. This should help to explain the high variability of clinical symptoms related to sensitization to nsLTPs in individual patients. Moreover, during food preparation and/or processing, it is important to take the localization of allergenic proteins into account. Exposure to food allergens depends on the localization of individual proteins within particular parts of edible plants, thus determining the type of clinical response, making the clinical picture of allergy to nsLTPs even more complex. Most LTPs are located in the peel and less in the pulp. However, in the case of some fruits and vegetables, seeds seem to be the most important. There are a few fruits and vegetables which are consumed with seeds, and those include tomato, kiwi, and pomegranate. Understanding the structure and biology of individual nsLTPs should help to solve many clinical problems caused by sensitization to those very potent allergens.

## 4. Materials and Methods

### 4.1. Protein Purification

Natural Act c 10.0101 was purified from seeds of ripe gold kiwifruit (*Actinidia chinensis*) purchased in a local market. Manually collected seeds were crushed by pestle in a mortar until a smooth powder was obtained. Proteins were extracted in 0.5 M NaCl, at 4 °C for 2 h and centrifuged at 10,400× *g* for 30 min. The supernatant was collected and used to purify Act c 10.0101 following an already reported procedure [63].

Pomegranate (*Punica granatum*) fruits were harvested at the commercial ripening stage. The arils contained in each pomegranate were manually separated into pulp and seeds. The red pulp was homogenized in a blender after the addition of 1M NaCl (1:1 *w*/*v* or *v*/*w*) and stirred at 4 °C for 2 h. Next, the sample was centrifuged at 17,300× *g* for 45 min and the supernatant was collected and used to purify Pun g 1 following a previously reported procedure [63]. The purity of Act d 10 and Pun g 1.0101 was checked by SDS-PAGE, Reverse Phase-HPLC, and N-terminal amino acid sequencing. Molar extinction coefficients at 280 nm of 3480 M^−1^ cm^−1^ and 4970 M^−1^ cm^−1^ were used to estimate the protein concentration of Act c 10.0101 and Pun g 1.0101 preparations, respectively.

### 4.2. Size-Exclusion Chromatography

The purified nsLTPs were subjected to size-exclusion chromatography using a fast protein liquid chromatography (FPLC) system, model AKTA pure 25L—Gold seal (GE Healthcare Europe GmbH, Milan, Italy). The proteins (0.1 mg) were loaded on a gel filtration column (Superdex 75 HR10/30; Amersham Biosciences, Uppsala, Sweden), equilibrated, and eluted with the selected buffer. The buffers used were: (i) 10 mM Tris-HCl, pH 8.0, 0.25 M NaCl; (ii) 50 mM sodium acetate, pH 4.6, 0.25 M NaCl; (iii) 50 mM sodium citrate pH 4.5, 0.25 M NaCl. The absorbance at 280 nm was recorded.

Both nsLTPs were eluted by gel filtration chromatography as a single peak, thus excluding the presence of multimeric forms (Supplementary Figure S1). In Tris-HCl buffer at pH 8.0, the proteins were eluted at exactly the same volume (14.41 mL) corresponding to a molecular mass of 7.24 kDa, a value lower than that calculated on the basis of the amino acid sequence. Pun g 1.0101 showed a molecular mass slightly higher (7.59 kDa) when eluted from gel filtration in sodium acetate, pH 4.6. Act c 10.0101 was eluted at a molecular mass of 9.54 kDa, a value similar to that obtained by mass spectrometry (9.46 kDa), when subjected to gel filtration in sodium citrate, pH 4.6 (Figure S1).

Therefore, on the basis of the elution volume on the precalibrated Superdex 75, the estimated molecular mass of the nsLTPs was consistent with a monomeric form of both proteins.

### 4.3. Mass Spectrometric Analysis

Mass spectrometric analysis was performed by LC-MS with the Q Exactive HF-X mass spectrometer and the ProSwift RP-4H (1 mm by 50 mm) HPLC column for accurate protein separation for small molecular weights (Thermo Scientific, Waltham, MA, USA).

### 4.4. Protein Crystallization and Data Collection

Protein crystals were obtained using the vapor diffusion technique. Experiments were performed using 96-well crystallization plates (Hampton Research, Aliso Viejo, CA, USA). Crystallization drops were set by mixing 1 µL of protein solution (1 mg/mL) with 1 µL of crystallization solution. Well-diffracting crystals for Act c 10.0101 were obtained using 0.1 M citric acid buffer, pH 4.0 with 3.0 M ammonium sulfate as the crystallization solution. Similarly, well-diffracting crystals for Pun g 1.0101 were obtained from 0.1 M sodium acetate, pH 4.6 and 2.0 M ammonium sulfate solution. X-ray diffraction experiments were carried out on crystals cryocooled in liquid nitrogen. Diffraction experiments were performed at the South East Regional Collaborative Access Team beamline 22ID at the Advanced Photon Source (Argonne National Laboratory, Lemont, IL, USA). HKL-2000 was used for data processing [64]. Data collection statistics are reported in Table 1.

### 4.5. Structure Determination, Refinement, and Validation

Both structures were solved by molecular replacement using MOLREP [65] integrated with HKL-3000 [66]. The structure of rice nsLTP (PDB code: 1RZL) was used as a search model for the molecular replacement procedure in the case of Act c 10.0101. For solving the Pun g 1.0101 structure, a structure of an nsLTP from eggplant (PDB code: 5TVI) was used. Initial models were rebuilt with ARP/wARP [67], BUCCANEER [68], COOT [69], and HKL-3000. Structure refinement was performed using REFMAC [70] and COOT. Local non-crystallographic symmetry was used during refinement, as implemented in REFMAC. TLS was implemented in the final stages of refinement, and the TLS Motion Determination server [71] was used to divide protein chains into segments. COOT and MOLPROBITY [72] were used for validation of the structures. Refinement and validation statistics are summarized in Table 1. Both Act c 10.0101 and Pun g 1.0101 structures with their structure factors were deposited to the PDB with accession codes 7KSB and 7KSC, respectively.

### 4.6. Various Computational Approaches

PDBePISA was used to investigate an oligomeric form of the proteins in crystal state [40]. CASTp was used for calculations of cavity volumes [73]. PDBeFOLD [74] and DALI [75] servers were used to find proteins with similar structures to Act c 10.0101 or Pun g 1.0101. Sequence alignment was prepared using Jalview [76] and MUSCLE [77]. The phylogenetic tree was prepared using Clustal Omega [78] and iTOL [79]. The figure showing protein structures was prepared with PYMOL [80]. The structure of Act d 10.0101 was modeled using I-Tasser [81] and the model of Act c 10.0101 as a template.

### 4.7. Calculations of A-RISC Indexes

A-RISC values were calculated as described previously [82]. Multiple sequence alignments of proteins belonging to the nsLTP family were performed with Clustal Omega [78]. Subsequently, sequence similarities and identities were calculated with SIAS with use of the default parameters (http://imed.med.ucm.es/Tools/sias.html). The A-RISC index for a pair of proteins is an average of the proteins’ sequence similarity and identity. The A-RISC index corresponds to a single numeric value that provides information on a relative homology between two proteins from the same family. Protein sequences of nsLTP allergens were obtained from Allergen Nomenclature [24] and Allergome [83] databases. nsLTPs that had complete sequences were used for calculations. A-RISC calculations were performed based on the mature versions of proteins with signal- and pro-peptides omitted.

## Figures and Tables

**Table 1:** Data collection and refinement statistics for Act c 10.0101 and Pun g 1.0101. Values in parentheses are for the highest resolution shell. RMSD—Root Mean Square Deviation.

Protein	Act c 10.0101	Pun g 1.0101
PDB accession code	7KSB	7KSC
**Data collection**		
Diffraction source	APS, 22ID	APS, 22ID
Wavelength (Å)	1.0000	1.0000
Space group	P2_1_	P2_1_
a, b, c, β (Å, °)	38.8, 38.1, 48.4, 98.9	29.5, 89.4, 58.9, 102.8
Resolution range (Å)	40.00–1.95 (1.98–1.95)	50.00–2.40 (2.44–2.40)
No. of unique reflections	10,217 (497)	10,593 (548)
Completeness (%)	98.7 (98.2)	90.6 (90.4)
Redundancy	4.0 (3.9)	2.8 (2.0)
<I/σ(I)>	25.1 (4.3)	6.6 (2.3)
R_meas_	0.113 (0.483)	0.220 (0.511)
R_p.i.m_	0.057 (0.240)	0.124 (0.316)
CC1/2	(0.904)	(0.751)
**Refinement**		
Resolution range (Å)	40.00–1.95 (2.00–1.95)	40.00–2.40 (2.46–2.40)
Completeness (%)	98.4 (92.7)	90.1 (85.0)
No. of reflections, working set	9707	10014
No. of reflections, test set	503	541
Final R*_cryst_*	0.199 (0.223)	0.204 (0.233)
Final R*_free_*	0.257 (0.380)	0.252 (0.348)
RMSD Bonds (Å)	0.013	0.010
RMSD Angles (°)	1.8	1.5
**Ramachandran Plot**		
Allowed regions (%)	100.0	100.0
Favored regions (%)	98.4	99.5

## Data Availability

Both Act c 10.0101 and Pun g 1.0101 structures with their structure factors were deposited to the PDB with accession codes 7KSB and 7KSC, respectively. Other data are available on request.

## Supplementary Materials

Figure S1: Gel filtration results, Figure S2: Superposition of Act c 10.0101crystal structure with model of Act d 10.0101.

## Sample Availability

Samples of the allergens (Act c 10.0101 and Pun g 1.0101) as well as raw diffraction images are available from the authors upon request.
